# Toothpicks

**Published:** 1882-11

**Authors:** 


### TOOTHPICKS.

The greatest chemist of modern times remarks that the civilization of a country or family is indicated by its consumption of soap. Perhaps as much may be said of the toothpick, for it is certain that America uses more toothpicks than do the effete monarchies and despotisms of the old world. A barbarian does not use toothpicks, either because his teeth are sound, which is the rare exception, or because he is a barbarian, which is the rule. A man who uses a toothpick is civilized. But there are degrees of civilization and refinement, and in order to find a scientific basis for the proper classification of toothpick using people, one might either use the material of the toothpick as a foundation, or the manner in which the toothpick is handled.

There are toothpicks of wood or quills, ivory or metal. The wooden toothpicks may be subdivided into those of hard and soft wood, of which the former are preferable. Then, again, a division may be based upon the point of the toothpick. Probably the best wooden toothpicks come from Japan; they are short, and can be used without being noticed. The quill toothpicks are either simple or ornamental. Ivory and golden toothpicks are a permanent article, while nearly all others are used once and then should be thrown away. Good toothpicks are more or less elastic, and nearly all of them are so cheap that everybody can afford to use them judiciously. A toothpick *per se* is strictly indifferent—that is to say, it has no moral or æsthetic value, the latter being bestowed by him or her who uses it. In this respect the toothpick resembles an act—it derives its dignity or vulgarity from the person behind it. And there lies the essence of the matter.

Imagine a lady in faultless attire, handsome and admirable, with a toothpick in her mouth. The spectacle is not pleasing. Or imagine a dinner party, the last course of which consists of toothpicks handed around by the waiters and then applied by the whole company. The sight is perfectly unpleasant. Every civilized man, woman and child has the right to use a toothpick, but have they the right to use toothpicks to the discomfort of others? And to fine organizations the visible use of the toothpick is a source of disgust. A man who uses a toothpick in public shows either that he is not aware of the annoyance which he gives to others or he defies good manners and prefers to be set down indelicate and gross. The same rule applies with double force to a woman, for women are the natural and conventional guardians of good manners. When sensible men are in doubt on a matter of politeness or fine propriety, they consult a lady. What, then, can be said of a lady who carries a toothpick in her mouth? She might as well rinse her mouth or brush her teeth in public.

The truth is that the associations of a toothpick are necessarily indelicate, for the toothpick reminds one of bad teeth or food particles held in the wrong place. The toothpick is, therefore, a toilet article, and ranks with the toothbrush, the nail cleaner or the ear spoon. These articles have to be used, but not in public. Every hand is marred by unclean finger-nails, but the nails ought not to be cleaned in public. Nor should teeth be brushed or picked in public. In hotel lobbies there are always men—not really gentlemen—and, alas! occasionally women, with a toothpick in their mouth. Quite likely these same persons eat with their knives and cut their finger-nails at the dinner table. But in this matter their example is not commendable.—*Boston Advertiser.*

